# Reply to ‘Comment on ‘MicroRNA-199b-5p attenuates TGF-β1-induced epithelial-mesenchymal transition in hepatocellular carcinoma’’

**DOI:** 10.1038/s41416-018-0031-z

**Published:** 2018-03-19

**Authors:** Shao-jun Zhou, Fu-yao Liu, Yuan-hui Jiang, Hui-fang Liang

**Affiliations:** 1 Department of General Surgery, Qilu Hospital of Shandong University, 107 West Culture Road, Jinan, 250012 China; 2 Department of General Surgery, Qilu Hospital of Shandong University, 758 Hefei Road, Qingdao, 266035 China; 3 Department of Gastrointestinal Medical Oncology, Division of Cancer Medicine, The University of Texas MD Anderson Cancer Center, 7455 Fannin Street, Houston, TX 77054 USA; 4 Research Laboratory and Hepatic Surgery Center, Department of Surgery, Tongji Hospital, Tongji Medical College, Huazhong University of Science and Technology, 1095 Jie Fang Da Dao, Wuhan, 430030 China

**Keywords:** Hepatocellular carcinoma, Hepatocellular carcinoma

We read with considerable interest the comments made by Cristóbal et al.^1^, in which they highlighted that SET deregulation could be playing a key role in the miR-199b/N-Cadherin interplay. We appreciate their thoughtful insights on the miR-199b-induced effects and their thoughts on the role of SET in cancer.

First, our study demonstrated that N-cadherin expression was markedly elevated in HCC. We investigated the epigenetic mechanisms of N-cadherin expression using computational and experimental approaches and we screened out miR-199b-5p, which directly targets N-cadherin in HCC. As Cristóbal et al. pointed out, miR-199b has been found to target SET, which plays a key role in miR-199b induced effects^2, 3^. Bioinformatic analysis and experiments have indicated that one miRNA may repress more than 100 mRNAs. Similarly, one mRNA may be targeted or regulated by quite a number of miRNAs^4, 5^. As previously reported, N-cadherin mRNA can be regulated using miR-145 and miR-124 in lung cancer^6, 7^. It has also been shown that miR-199b directly targets hypoxia-inducible factor 1α (HIF-1α) and SIRT1 in prostate and colon cancer, respectively^8, 9^. Therefore, miR-199b can exert its functionality by regulating a considerable number of targeted genes; however, its effects can only be partially reversed by a single gene.

Second, during our study, miR-199b was found to be involved in TGF-β-induced epithelial mesenchymal transition (EMT) in HCC. SET, as Cristóbal et al. pointed out, has been shown to play a role in EMT in pancreatic cancer through N-Cadherin regulation^10^. Similarly, HIF-1α and SIRT1, as target genes of miR-199b, have been found to trigger EMT by regulating N-Cadherin in cancer cells^11–14^. It is implied that miR-199b could regulate multiple pathways to affect TGF-β-induced EMT in HCC. The precise mechanism underlying TGF-β induced EMT remain unclear. Additional experimental studies are needed to explore and demonstrate which type of regulation is dominant.

Finally, we discovered that miR-199b suppresses both migration and invasion, and reduces TGF-β-induced Akt phosphorylation in HCC. A positive regulatory loop between N-cadherin and Akt signalling has been found as well. We are grateful to Cristóbal et al. for their insights on the possible molecular mechanisms involved in TGF-β-induced Akt phosphorylation. The interaction between miR-199b/N-Cadherin and Akt signaling needs to be further investigated to unveil the mechanisms of TGF-β-mediated EMT in HCC cells.
